# The Effect of Nutrient Supplementation on Female Fertility: A Systematic Review

**DOI:** 10.7759/cureus.67028

**Published:** 2024-08-16

**Authors:** Ahlam S Alrashidi, Lujain Feraih Aljaghwani, Raghad Saleh AlMohimeed

**Affiliations:** 1 Department of Obstetrics and Gynecology, Maternity and Children Hospital, Buraydah, SAU

**Keywords:** reproductive health, supplement, antioxidant, vitamin, female fertility

## Abstract

Assisted reproductive technologies (ART) have improved infertility treatment but reproductive outcomes remain challenging. Nutrient supplementation is being explored to enhance pregnancy rates, increase live birth rates, and reduce miscarriage rates in females undergoing ART. Nutrients like folic acid, omega-3 fatty acids, and antioxidants have shown potential benefits, yet conflicting results exist. Live birth rates may also be influenced by nutrient supplementation, with coenzyme Q10 and vitamin D showing promise. Miscarriage rates may be reduced with nutrients such as vitamin D, omega-3 fatty acids, and antioxidants, although more research is needed for definitive conclusions. Scientific and medical literature databases such as Cochrane Library, PubMed, and Web of Science were queried to identify relevant English publications adhering to predetermined inclusion and exclusion criteria. Various reproductive metrics, encompassing biochemical pregnancy rate, clinical pregnancy rate, ongoing pregnancy rate, implantation rate, live birth rates, and miscarriage rates, were assessed as clinical endpoints. The study population included 996 female subjects receiving ART. Two studies performed investigations on subjects diagnosed with unexplained infertility, two studies specifically included polycystic ovary syndrome patients, and five studies did not provide any specific information on the type of infertility or subfertility. All studies reported on the clinical/ongoing pregnancy rate, among which four included studies observed a significantly higher rate. Out of the four studies that reported on implantation rates, three found significantly higher rates in treatment groups. Out of the three studies that reported on biochemical pregnancy rates, two studies found significantly higher rates in treatment groups. With respect to the clinical outcomes that were studied in this analysis, variable effects of nutritional supplementation on reproductive parameters were observed. Some studies reported significantly higher rates of clinical/ongoing pregnancy, implantation, biochemical pregnancy, and live birth, while no significant difference was found in miscarriage rates.

## Introduction and background

Assisted reproductive technologies (ART) have revolutionized the field of reproductive medicine, offering hope for couples facing infertility. Despite advancements in ART procedures, achieving successful reproductive outcomes remains a complex challenge. Suboptimal embryo implantation, low pregnancy rates, and increased rates of miscarriage continue to be major concerns [1]. In recent years, researchers have turned their attention to exploring the role of nutrient supplementation in improving reproductive outcomes for females undergoing ART procedures [2-5]. This study aimed to critically examine the impact of nutrient supplementation on pregnancy rates, live birth rates, and miscarriage rates in females undergoing ART.

Infertility affects a substantial proportion of the population worldwide, with ART serving as a common treatment option. Nutrient supplementation has gained attention as a potential intervention strategy due to its influence on various physiological processes critical to reproductive success. Studies have highlighted the importance of optimal nutrition in maintaining hormonal balance, improving oocyte quality, and supporting endometrial receptivity, all of which are essential for successful conception and pregnancy (Table 1) [3-8].

**Table 1:** Algorithm for nutritional supplementation in patients receiving assisted reproductive technologies (ART). The permission was obtained from the original publishers to reproduce the content of this table [8].

Step	Action
Evaluate patient profile	Review medical history, current medications, fertility treatments, nutritional status, and lifestyle factors.
Assess specific nutritional needs	Identify deficiencies through clinical assessments and tests. Focus on key nutrients like folic acid, omega-3s, antioxidants, vitamin D, CoQ10, and iron.
Develop supplementation plan	Collaborate with a fertility specialist or dietitian to tailor a plan that considers patient needs and potential interactions.
Determine dosages and timing	Set dosages based on guidelines and research. Optimize timing for preconception, ovarian stimulation, or throughout the ART cycle.
Educate and counsel patient	Explain the purpose, benefits, and risks of supplements. Provide administration guidance, including dosage and timing.
Monitor and adjust	Track progress with follow-up visits and tests. Adjust the plan based on patient response and outcomes.
Provide ongoing support	Offer continuous counseling, address concerns, and encourage adherence to the plan alongside a balanced diet.
Collaborate with fertility team	Ensure communication with the fertility specialist and team for integrated care and updates on the supplementation plan.

One of the primary areas of investigation regarding nutrient supplementation in ART is its effect on pregnancy rates. Several nutrients, such as folic acid (FA), omega-3 fatty acids, and antioxidants, have been associated with improved pregnancy outcomes [4,7,9-14]. For instance, FA plays a crucial role in DNA synthesis and methylation, affecting oocyte and embryo development [11]. Omega-3 fatty acids exhibit anti-inflammatory properties, potentially enhancing endometrial receptivity [7]. Antioxidants, including vitamins C and E, have been linked to reduced oxidative stress, thereby promoting embryo development and implantation [15,16]. However, conflicting results have been reported, necessitating a thorough examination of the existing evidence.

Furthermore, live birth rates are another crucial outcome measure in assessing the efficacy of nutrient supplementation in ART [17]. Some studies have reported positive associations between certain nutrients and live birth rates. For example, coenzyme Q10 has been suggested to enhance oocyte quality, while vitamin D has been linked to improved endometrial receptivity [5,18]. These findings underscore the potential benefits of nutrient supplementation in increasing the chances of a successful live birth. However, additional research is needed to establish robust conclusions due to variations in study design, sample sizes, and patient characteristics.

Miscarriage rates represent a significant concern for couples undergoing ART, with elevated rates compared to natural conception. Nutrient deficiencies and oxidative stress have been implicated in miscarriages, prompting investigations into the potential role of nutrient supplementation in reducing miscarriage rates [19,20]. Preliminary studies suggest that specific nutrients, such as vitamin D, omega-3 fatty acids, and antioxidants, may confer protective effects against miscarriages [21]. However, the available evidence is limited and warrants further investigation to establish causal relationships.

## Review

Methods

Definition of Outcomes and Inclusion Criteria

Studies determining reproductive outcomes following nutritional supplementation in female ART patients were included. The following clinical outcomes were reported by the studies: biochemical pregnancy rate, clinical pregnancy rate, ongoing pregnancy rate, implantation rate, live birth rates, and miscarriage rates.

To be included in this systematic review, studies had to meet the following inclusion criteria: (1) involved female patients undergoing ART, (2) investigated the impact of nutritional supplementation on reproductive outcomes, specifically reporting biochemical pregnancy rate, clinical pregnancy rate, ongoing pregnancy rate, implantation rate, live birth rates, or miscarriage rates, and (3) were original research articles (randomized controlled trials, cross-sectional, case-control, or cohort studies) published in English. The exclusion criteria were as follows: (1) non-human or in vitro studies, (2) reviews, commentaries, opinion pieces, editorials, or case reports, (3) studies including male participants or mixed-gender populations without separate analysis for female participants, (4) studies focusing on populations with unrelated medical conditions, (5) studies not specifically addressing nutritional supplementation or involving non-nutritional interventions, (6) studies not reporting relevant reproductive outcomes, (7) non-English studies without reliable translations, and (8) studies published prior to 2000. This rigorous selection process ensured the inclusion of high-quality, relevant studies that provided robust evidence on the effect of nutrient supplementation on female fertility outcomes.

Search strategy

Scientific literature databases such as PubMed, Web of Science, and Cochrane Library were systematically queried to identify articles meeting predetermined inclusion and exclusion criteria. The search strategy utilized keywords including "female fertility," "vitamin," "folic acid," "folate," "iron," "vitamin D," "zinc," "antioxidant," "omega-3 fatty acids," "mineral," "nutrient," and "supplement." Additional terms such as "reproductive health," "conception," "ovulation," "ovarian reserve," "egg quality," "birth rate," "miscarriage," and "implantation" were sequentially incorporated into the search process to enhance its comprehensiveness. Furthermore, relevant publications were identified by reviewing the reference lists of retrieved articles. The search was limited to studies published between 2003 and 2023 to ensure the inclusion of the most relevant and recent research findings.

Screening and extraction

Articles with irrelevant titles, such as those focusing on male fertility or unrelated medical conditions (e.g., cancer treatment), were excluded from consideration. For example, titles like "Male infertility: role of vitamin D and oxidative stress markers" or "Use and effects of oral nutritional supplements in patients with cancer" were excluded. In the subsequent phase, both the full text and abstracts of the papers were meticulously reviewed to determine their compliance with the inclusion criteria. To streamline the process, titles and abstracts were organized, assessed, and scrutinized for duplicates using EndNote X8 (Philadelphia, PA: Clarivate Analytics). To ensure the highest quality of selection, a dual screening approach was adopted, involving one screening for the evaluation of titles and abstracts, and another for the comprehensive examination of the entire texts. Once all relevant articles were identified, a structured extraction sheet was created to capture pertinent information aligned with our specific objectives. This included delineating the desired outcomes and baseline characteristics such as study design, the total number of patients undergoing ART, the method of ART employed, the type of nutritional supplements utilized, and the geographical location of the study.

Quality assessment

The Jadad scale scoring method was utilized to assess the methodological quality of the included randomized controlled trials (RCTs) [22]. Research investigations are evaluated based on the inclusion of three critical methodological components in clinical trials as follows: randomization, blinding, and the accountability of all participants, including those lost to follow-up or withdrawal. Each affirmative response to the initial five criteria adds one point, whereas a positive response to either of the final two criteria deducts one point, resulting in a scoring range of 0-5. Scores between 0 and 2 signify low quality, while scores ranging from 3 to 5 denote high quality.

For cross-sectional, case-control, and cohort studies, we used the modified Newcastle-Ottawa scale (NOS), which consists of three primary domains as follows: the quality of methods, compatibility, assessment, and reporting of the results [23]. Each category is assigned a maximum of five, two, and three stars, respectively, on this rating scale. Power calculations, sequential participant selection, and recruitment bias were employed to assess selection bias. Studies that accounted for participant age, Body Mass Index (BMI), or other variables such as polycystic ovary syndrome (PCOS) and unexplained infertility were utilized to ensure comparability. It is widely accepted that research exhibiting minimal bias should be awarded a maximum of five stars. Studies were graded on a scale of 0-10, with scores categorizing them as poor (0-4), satisfactory (5, 6), good (7, 8), or very good (9, 10).

Results

Search Results

**Figure 1:**
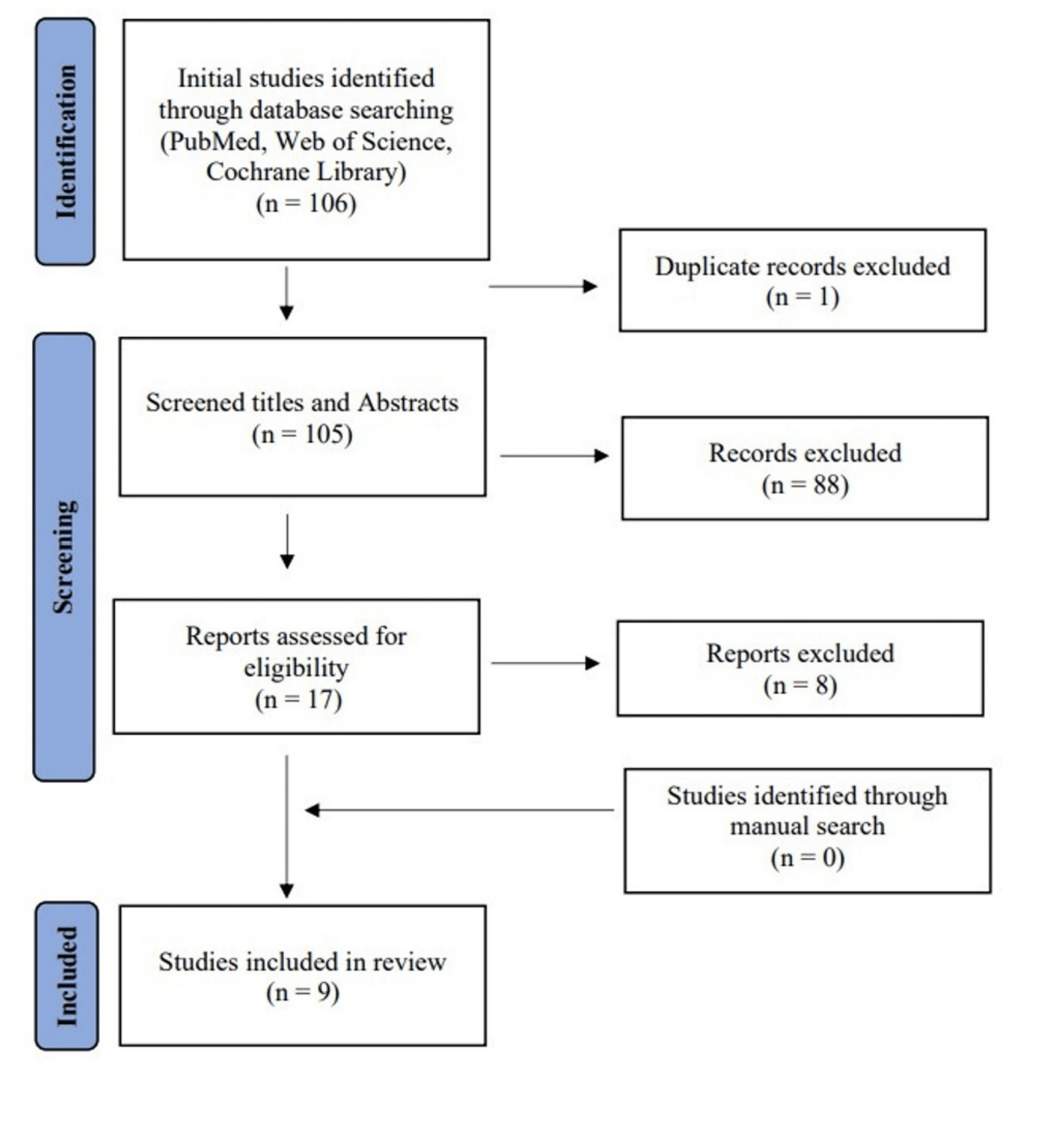
Preferred Reporting Items for Systematic Reviews and Meta-Analyses (PRISMA) flow diagram.

Utilizing the prescribed search methods, we identified a sum of 106 citations, which decreased to 105 following the removal of duplicate entries. Subsequent screening of titles and abstracts led to the retention of only 17 citations, which progressed to the subsequent stages. Following a thorough examination of the full texts, only nine articles were found to meet our predetermined inclusion and exclusion criteria. Figure 1 displays the thorough search and screening procedure.

Results of Quality Assessment

Most of the incorporated studies exhibited satisfactory or superior quality with minimal risk of bias, as determined by our bias assessment. None of the mentioned research investigations yielded unsatisfactory results (Table 2) [24-29].

**Table 2:** Modified Jadad scores of the included randomized control trials.

Studies	Was the research described as randomized?	Was the approach of randomization appropriate?	Was the research described as blinded?	Was the approach of blinding appropriate?	Was there a presentation of withdrawals and dropouts?	Was there a presentation of the inclusion/exclusion criteria?	Was the approach used to assess adverse effects described?	Was the approach of statistical analysis described?	Total
Morsy et al. [24]	1	1	0	0	1	1	0	1	5
Fatemi et al. [25]	1	1	1	1	1	0	1	1	7
Doryanizadeh et al. [26]	0	0	1	1	1	1	0	0	4
Cicek et al. [27]	1	1	0	0	1	1	0	1	5
Espinola et al. [28]	0	1	0	0	0	1	0	1	3
Agrawal et al. [29]	1	1	1	1	1	1	1	1	8

Characteristics of the study included

Finally, we evaluated a total of nine studies that satisfied the eligibility criteria of this investigation and hence are included in this systematic review. The studies were conducted between 2003 and 2021 and included 996 females receiving ART, among which 485 subjects were in the treatment arm or group, and 511 controls. Based on the studies that reported on the participant ages, the range of age was determined to be between 18 and 42 years. Six of the included research investigations were RCTs [23-28], followed by each cohort study [29], case-control study [30], and a cross-sectional study [31]; the data among all studies was prospectively collected. Regarding the geographical distribution of the included studies, Italy [27,31] and Iran were the subject of two studies each [24,25], while the Czech Republic [30], Turkey [26], Egypt [23], the United Kingdom [28], and the United States were each represented by a single study [29]. Two studies performed investigations on subjects diagnosed with unexplained infertility [26], two studies specifically included PCOS patients [24], and five studies did not provide any specific information on the type of infertility or subfertility [27,29-32]. The main characteristics of the included studies have been summarized in Tables 3, 4.

**Table 3:** Summary of the results of bias assessment using the Newcastle-Ottawa scale of each included study.

Studies	Study type	Selection	Comparability	Outcome	Total quality score
Gaskins et al. [30]	Cohort study	4	2	2	8
Crha et al. [31]	Case-control study	4	2	2	8
Paffoni et al. [32]	Cross-sectional study	2	2	3	7

**Table 4:** Baseline characteristics of the studies included in this review. ART: assisted reproductive technology; IVF: in vitro fertilization; ET: embryonic transfer; RCT: randomized controlled trial; hCG: human chorionic gonadotropin; ICSI: intracytoplasmic sperm injection

Studies	Year	Country	Study design	Recruited patients (n)		ART method	Patient age range	Type of nutrient supplement	Duration of nutrient supplement
				Treatment	Control				
Morsy et al. [24]	2020	Egypt	RCT	30	30	-	20-28 years	Vitamin E and D3	Start to the end of the study
Fatemi et al. [25]	2017	Iran	RCT	44	46	ICSI	18-38 years	Vitamin E and vitamin D3	8 weeks
Doryanizadeh et al. [26]	2021	Iran	RCT	36	38	IVF	27-36 years	Calcitriol	Daily for 4 weeks
Cicek et al. [27]	2012	Turkey	RCT	53	50	Controlled ovarian stimulation and intrauterine insemination	18-37 years	Vitamin E	3rd to the 5th day of the menstrual cycle until hCG injection day of the controlled ovarian stimulation
Espinola et al. [28]	2021	Italy	RCT	50	50	IVF	22-42 years	Vitamin D3	12 weeks
Agrawal et al. [29]	2012	UK	RCT	30	28	Standard treatment regimens with clomiphene citrate/gonadotropins	19-40 years	Multiple micronutrient supplements and folic acid	Daily until the study is completed
Gaskins et al. [30]	2015	USA	Cohort study	50	50	ICSI	30-38 years	Serum folate and vitamin B-12	-
Crha et al. [31]	2003	Czech Republic	Prospective case-control	38	38	IVF/ET	-	-	-
Paffoni et al. [32]	2014	Italy	Cross-sectional study	154	181	IVF	32-41 years	-	-

The major study endpoints such as biochemical pregnancy rate, clinical/ongoing pregnancy rate, implantation rate, live birth rates, and miscarriage rates have been defined in Table 5 and summarized in Table 6.

**Table 5:** Definition of terms related to pregnancy outcomes. The permission was obtained from the original publishers to reproduce the content of this table [33-38].

Pregnancy outcome terms	Definitions
Biochemical pregnancy rate	The rate of pregnancies is confirmed by detecting the presence of pregnancy hormones, such as human chorionic gonadotropin (hCG), in the blood or urine. It indicates the early stage of pregnancy, typically before the pregnancy can be visualized through ultrasound or confirmed by other clinical signs.
Clinical pregnancy rate	The rate of pregnancies confirmed by clinical evaluation, usually through ultrasound imaging, shows the presence of a gestational sac or an embryo/fetus within the uterus. This assessment verifies the viability and location of the pregnancy and confirms that it is progressing as expected.
Ongoing pregnancy rate	The rate of pregnancies that have successfully progressed beyond the early stages and are still ongoing at a specific point in time, typically measured after a certain gestational age (e.g., 12 weeks). It represents the likelihood of the pregnancy continuing to a live birth.
Implantation rate	The rate at which embryos successfully implant into the uterine lining after transfer during assisted reproductive technologies (ART), such as in vitro fertilization (IVF). It indicates the proportion of embryos that establish a connection with the uterus and have the potential to develop further.
Live birth rate	The rate of pregnancies that result in the birth of a living infant. It represents the ultimate outcome of a successful pregnancy, accounting for the survival of the fetus through gestation and delivery. It is an essential measure of reproductive success in assessing the effectiveness of fertility treatments or interventions.
Miscarriage rate	The rate of pregnancy loss before the fetus reaches the stage of viability, typically defined as before 20-24 weeks of gestation or when the fetus weighs less than 500 g. It represents the frequency of spontaneous termination of a pregnancy and is an important factor to consider in evaluating reproductive outcomes and the success of fertility treatments.

**Table 6:** Summary of the outcomes of the studies included in this review. NS: not significant; Q: quartile; FA: folic acid; Vit B12: vitamin B-12

Studies	Biochemical pregnancy rate	Clinical/ongoing pregnancy rate	Implantation rate	Live birth rate	Miscarriage rate
Morsy et al. [24]	-	4.5% vs 7%, p=0.491	-	-	
Fatemi et al. [25]	69% vs 25.8%, p<0.001	62.1% vs 22.6%, p=0.002	35.05% vs 8.6%, p<0.001	-	-
Doryanizadeh et al. [26]	31.4% vs 18.2%, p=0.032	25.5% vs 13.6%, NS	-	-	19.6% vs 6.8%, NS
Cicek et al. [27]	-	18.9% vs 14%, NS	18.9% vs 16%, NS	-	-
Espinola et al. [28]	4.5% vs 6.3%, NS	81.8% vs 75%, NS	37.1% vs 19.2%, p=0.015	-	13.6% vs 18.8%, NS
Agrawal et al. [29]	-	66.7% vs 39.3%, p=0.013	-	-	0.03% vs 0.14%, NS
Gaskins et al. [30]	-	Q4 vs Q1, FA: 60% vs 40%, p=0.04; Vit B12: 66.7 % vs 46.5 %, p=0.01	Q4 vs Q1, FA: 62.9% vs 48.9%, p=0.26; Vit B12: 72.7 % vs 52.1%, p=0.03	Q4 vs Q1, FA: 51.4% vs 31.1%, p=0.01; Vit B12: 60.6% vs 27.9 %, p=0.008	-
Crha et al. [31]	-	34.2% vs 23.7%, NS	-	-	-
Paffoni et al. [32]	-	31% vs 20%, p=0.02	21% vs 13%, p=0.006	-	-

All studies reported on the clinical/ongoing pregnancy rate, of which four included studies observed a significantly higher rate. Out of the four studies that reported on implantation rates, three found significantly higher rates in treatment groups [25,28,32]. Out of the three studies that reported on biochemical pregnancy rates, two studies found significantly higher rates in treatment groups [25,26]. One study reported on the live birth rate, and here, the rate was significantly higher in the treatment group [30]. None of the three studies that reported on miscarriage rates found a statistical difference between treatment and control groups [26,28,29]. Overall results showed variable effects of nutritional supplementation on reproductive parameters.

Discussion

Multiple Micronutrients

Arhin et al. conducted a systematic review of the effect of micronutrient supplementation on in vitro fertilization (IVF) outcomes in 2017 [39]. Micronutrients were defined as "essential dietary elements or organic compounds that are required in only small quantities for normal physiologic processes to occur." They methodically synthesized clinical data concerning the impact of micronutrient intake on the primary outcome measures of in vitro fertilization (IVF) treatment. In this investigation, the primary clinical endpoints of interest were the fertilization rate (defined as the number of fertilized oocytes per number of oocytes injected) and pregnancy rate (determined by the presence of a gestational sac observed via transvaginal ultrasound). The analysis revealed that supplementation with single or multiple micronutrients (MMN), including antioxidant therapy, may exert an influence on key clinical outcomes in couples undergoing IVF treatment, such as pregnancy and live birth rates.

In the present systematic review, Agrawal et al. looked at whether ovulation induction (OI) with clomiphene citrate (CC)/gonadotrophins in subfertile female with adjuvant MMN dietary supplementation led to higher pregnancy rates compared to FA alone at a teaching hospital. Adjuvant MMN supplements, or FA, were given to females undergoing OI [29]. Following the completion of the third treatment cycle or upon conception, the clinical pregnancy rates and blood nutrient levels were assessed. Analysis conducted according to the intention-to-treat principle demonstrated that females supplemented with adjunct MMN had a notably higher cumulative clinical pregnancy rate (66.7%) compared to those receiving FA alone (39.3%; p=0.013). Moreover, the ongoing pregnancy rate among females receiving MMN supplementation was 60.0% vs 25.0% in the FA group (p=0.02). Additionally, females supplemented with MMN required significantly fewer attempts to achieve pregnancy compared to those receiving FA alone (p<0.001). The authors concluded that females who take adjuvant MMN supplementation during OI may have a higher chance of pregnancy compared with females on FA. Females taking MMN supplements had a 60.0% continuing pregnancy rate compared to a 25.0% rate (p=0.02) in the FA group. Additionally, compared to females using FA, females taking MMN supplements took considerably fewer attempts to get pregnant (p<0.001). The scientists concluded that, compared to females taking FA, those taking adjuvant MMN supplementation through OI may have a larger likelihood of becoming pregnant.

Vitamin D

Cozzolino et al. conducted a systematic review and meta-analysis to evaluate the relationship between serum levels of 25-hydroxy vitamin D [25(OH)D] and the outcomes of IVF in infertile females in 2020 [40]. They included studies comparing IVF outcomes between infertile females with vitamin D levels below 20 ng/mL and those with levels equal to or above 20 ng/mL. The findings revealed that deficient vitamin D levels were not significantly associated with a higher risk of lower clinical pregnancy rates (risk ratio (RR): 0.88; 95% confidence interval, CI: 0.69-1.11). However, lower vitamin D status was linked to a lower rate of live births (RR: 0.76, 95% CI: 0.61-0.93). Further, Seko et al. conducted a systematic review and meta-analysis focused on investigating the impact of melatonin supplementation during controlled ovarian stimulation (COS) on the outcomes of ART in females [41]. None of the five included studies provided information on the live birth rate or congenital abnormalities. The calculated risk ratio of 1.21 with a 95% confidence interval ranging from 0.98 to 1.50 indicated that there was no statistically significant increase in clinical pregnancy rate associated with melatonin supplementation. Similarly, the findings regarding miscarriage rates and interventions to mitigate ovarian hyperstimulation syndrome (OHSS) were also inconclusive due to imprecise estimates.

In the present systematic review, Espinola et al. examined the effects of oral vitamin D3, myo-inositol, FA, and melatonin supplementation on the success of IVF [28]. Twenty consecutive infertile females receiving IVF therapy were divided into two groups by a 1:1 random distribution. Myo-inositol, alpha-lactalbumin, and FA were given to the females in group A (the control) in the morning, and myo-inositol, FA, and melatonin were given to them in the evening. Females in group B (treated) were given a similar regimen, but they also received cholecalciferol (vitamin D3) every evening starting at the onset of the luteal phase. The statistical study took into account the 50 patients from group A and the 50 patients from group B who underwent blastocyst transfer. After 45 days of treatment, vitamin D3 levels considerably rose as follows: 33.2 ng/mL in group B vs 24.3 ng/mL in group A (p<0.001). Additionally, the implantation rate rose to 37.1% in group B against 19.2% in group A (p=0.0151). The authors came to the conclusion that higher levels of vitamin D3 were positively related to the success rate of IVF.

The aim of the study by Doryanizadeh et al., which is also included in this review, was to investigate how utilizing calcitriol, an active form of vitamin D, affected the outcomes of IVF in females who were vitamin D deficient [26]. A total of 180 infertile females who received IVF treatment participated in this double-blinded, randomized clinical experiment. Ninety-five of them were discovered to have low levels of vitamin D (blood serum 25-dihydroxy vitamin D <30 ng/mL). Forty-four participants received a placebo, whereas 51 females in the experimental group received two 0.25 g calcitriol pills daily for four weeks, which were discontinued 8 hours before the embryo transfer (mean vitamin D insufficiency: 27.5±1.8 ng/mL in the case group vs. 27.6±1.8 ng/mL in the control group, p>0.05). The results of chemical and clinical pregnancy in 74 females (including 36 in the case group and 38 in the control group) were included in the final analysis. The study found that, at 31.4 vs. 18.2% (p>0.05), chemical pregnancy success was considerably higher in the treatment group than in the control group. Reaching the clinical pregnancy stage (25.5% in the case group vs. 13.6% in the control group) and continuing the pregnancy into week 20 (9.8% in the case group vs. 11.6% in the control group) did not differ significantly between both groups (p>0.05). The investigators came to the conclusion that administering calcitriol to infertile females with vitamin D insufficiency can considerably boost the likelihood that an IVF cycle will be successful by enhancing the course of implantation.

Through a prospective cross-sectional study in an infertility clinic of a teaching hospital, Paffoni et al. looked at the success rates of IVF in females with low serum levels of 25-hydroxyvitamin D [25(OH)D] (20 ng/mL) [32]. An age range of 18-42 years, a BMI of 18-25 kg/m^2^, and an acceptable ovarian reserve as determined by the Bologna criteria were the primary inclusion criteria. At the time of cycle preparation, eligible females were given a serum sample for 25(OH)D testing. If the cycle was postponed or the attempt took an unusually long time, subjects were excluded from the study. Quantitative serum 25(OH)D measurement was the intervention. The clinical pregnancy rate was the key outcome parameter. There were 154 and 181 recruited females with serum 25(OH)D levels below and above 20 ng/mL, respectively. The adjusted odds ratio for clinical pregnancy in females with vitamin D levels below 20 ng/mL was 2.15 (95% CI: 1.23-3.77), and the clinical pregnancy rates were 20% (30/154) and 31% (56/181), respectively (p=0.02). According to subgroup analysis, the group of females with the highest serum levels (>30 ng/mL) had the greatest likelihood of becoming pregnant. The authors concluded that vitamin D is an additional element that affects female fertility and the success of IVF.

Vitamin E

Wu et al. conducted a systematic review and meta-analysis that investigated how vitamin E supplementation affected the thickness of the endometrium and pregnancy outcomes in females facing infertility [42]. It was discovered that, on average, the endometrium in the vitamin E therapy group was thicker than that in the control group (SMD=0.57, 95% CI: 0.26, 0.87, p=0.0002). A subgroup analysis revealed no discernible difference across daily doses of either 100 mg or 400 IU of vitamin E. With or without vitamin E, the risk of ongoing pregnancies did not differ significantly (OR=1.08, 95% CI: 0.72, 1.62, p=0.70). The available data suggests that females of reproductive age who use vitamin E supplements may have thicker endometriums.

In the present systematic review, Cicek et al. researched the impact of vitamin E on the outcomes of controlled ovarian stimulation and intrauterine insemination (IUI) for females with unexplained infertility [27]. The control group (group B, n=50) experienced OI without vitamin E, while the study group (group A, n=53) underwent controlled ovarian stimulation with CC and 400 IU/day p.o. of vitamin E delivery. The groups' therapeutic results were assessed. Regarding the demographic results, there were no noteworthy variations between the two groups. There was a significant difference in endometrial thickness between the two groups on the day when hCG was administered (p<0.001). Odds ratios and the associated 95% confidence interval were calculated to determine the impact of vitamin E supplementation on the percentages of implantation and continued pregnancy. Receiving vitamin E had no statistically significant effect on the rates of implantation or continuing pregnancy, with ORs of 1.22 and 1.43, respectively, and 95% confidence intervals of 0.44 and 4.1. The scientists concluded that vitamin E supplementation may enhance the endometrial response in females who are infertile for unknown reasons due to its probable antioxidant and anticoagulant properties. Additionally, it may regulate the issue of a thin endometrium throughout these cycles and regulate the antiestrogenic effects of CC.

Morsy et al.’s study in this systematic review examined the impact of vitamin E on ovulation and conception in females with PCOS who were resistant to CC [24]. On females with CC-resistant PCOS, prospective, randomized, controlled, open-label research was conducted. In the control group (n=30), metformin 500 mg was given three times daily in addition to 150 mg of CC for five days beginning on the third day of menstruation for three menstrual cycles. In the group receiving vitamin E (n=30), a daily dosage of 1500 IU of vitamin E was administered throughout the study alongside metformin and CC according to the established schedule. The primary endpoint assessed was the cumulative ovulation rate, while secondary endpoints included the pregnancy rate, serum midluteal progesterone levels, mean follicular diameter, quantity of dominant follicles, and endometrial thickness. Pregnancy occurred in four (4.5%) of 88 cycles in the control group and six (7%) of 86 cycles in the vitamin E group (p=0.491), with ovulation observed in 57 (64.8%) of 88 cycles in the control group and 63 (73.3%) of 86 cycles in the vitamin E group. There were no significant differences between groups in serum midluteal progesterone levels, the number of dominant follicles, or mean follicular diameter. However, the endometrium was notably thicker in the vitamin E group compared to the control group. The findings of the trial did not support the authors' hypothesis that vitamin E supplementation would enhance ovulation and pregnancy rates in CC-resistant PCOS patients.

Vitamin D and E

In this systematic review, the study by Fatemi et al. looked at the potential impact of coupled vitamin E and D intake on the outcomes of PCOS patients undergoing intracytoplasmic sperm injection (ICSI) (oocyte number and quality, embryo number and quality, and pregnancy rate) [25]. A total of 105 infertile females diagnosed with PCOS and scheduled for intracytoplasmic sperm injection (ICSI) were enrolled in a double-blinded trial. They were randomly assigned to receive either the intervention group (vitamin E at 400 mg/day and vitamin D3 at 50,000 IU once every two weeks) or the placebo group for a duration of eight weeks. The primary outcomes assessed were implantation rate, pregnancy rate, and clinical pregnancy rate, while secondary outcomes included oocyte quality, embryo quality, and fertilization rate. Additionally, the study investigated the associations between total antioxidant capacity (TAC), serum vitamin D3 levels, and levels of malondialdehyde (MDA) in follicular fluid and blood. The intervention group demonstrated significantly higher implantation rate, pregnancy rate, and clinical pregnancy rate compared to the placebo group (p<0.001). Analysis revealed a substantial increase in serum MDA levels and a significant decrease in serum TAC levels in both groups post-treatment compared to baseline. Further examination showed a weak yet significant correlation between vitamin D status and implantation rate (p=0.015) as well as improved clinical pregnancy (p=0.037). However, no significant association was found between TAC and ICSI outcomes, either in follicular fluid or serum MDA levels. Ultimately, the study findings did not provide additional clinical evidence supporting the notion that vitamins E and D3 may influence the success rate of IVF through an antioxidant mechanism.

Vitamin C

In their literature review, Ruder et al. examined probable causes of oxidative stress from the ovarian germ cell through the stages of human pregnancy and pregnancy difficulties associated with infertility [43]. They also evaluated the epidemiology of female infertility related to antioxidant defenses and oxidation. It has been suggested that female oxidative stress is probably a mediating factor in conception and that there are thresholds for oxidative stress depending on anatomical location and preconceptional stage.

Included in our systematic review is a prospective study by Crha et al., in which the impact of vitamin C supplementation on infertility treatment outcomes in females undergoing in vitro fertilization and embryonic transfer (IVF/ET) is examined [31]. The study included 76 females, divided into smokers and non-smokers, with half of them receiving daily doses of 500 mg of vitamin C. Ascorbic acid levels in the follicular fluid were measured using a colorimetric method in two urine samples as follows: one taken before vitamin C supplementation and the other at follicle retrieval. Ovarian response to hormonal stimulation, fertilization rates, and embryo development were evaluated based on the number of follicles formed, retrieved oocytes, successfully fertilized oocytes, and developed embryos. The effectiveness of infertility treatment was assessed by the number of pregnancies achieved. The results showed that females using vitamin C supplements had significantly higher ascorbic acid levels in their follicles compared to the control group. However, the administration of vitamin C during hormonal stimulation did not have a statistically significant effect on the number of pregnancies overall. Nonetheless, in the non-smokers' group, vitamin C supplementation led to an increased number of pregnancies. The study also found that non-smoking females had significantly higher pregnancy rates compared to smokers, emphasizing the importance of smoking cessation before infertility treatment.

Strength and limitations

This systematic review provides a thorough analysis of the impact of nutrient supplementation on reproductive outcomes. However, our review does have certain limitations that need to be acknowledged. Firstly, there is heterogeneity among the included studies regarding the specific type and dosage of micronutrients, the methods of assisted reproductive technology employed, and the timing of nutrient supplementation (whether it was before IVF treatment, during ovarian stimulation, or at the time of oocyte retrieval). Furthermore, not all of the studies included in our analysis were RCTs, and there was a potential bias in the selection of the participants studied. Moreover, the availability of data on live birth rates, which is the most significant patient-centered outcome, was limited to only one study.

## Conclusions

Nutrient supplementation represents a promising avenue for improving reproductive outcomes in females undergoing ART procedures. While certain nutrients such as vitamin D have shown potential benefits in enhancing pregnancy rates, live birth rates, and reducing miscarriage rates, the evidence remains inconclusive. Variability in study methodologies and participant characteristics underscore the need for well-designed, large-scale clinical trials to provide definitive conclusions. Understanding the impact of nutrient supplementation on reproductive outcomes can offer valuable insights into optimizing ART procedures and improving the success rates for couples facing infertility.
